# 

**Published:** 1868-06-01

**Authors:** 


Vol. XXV. Number 11.

THE

### Chicago Medical Journal.

*PUBLISHED SEMI-MONTHLY.*

EDITED BY

J. Adams Allen, M.D., LL.D.

JUNE 1, 1868.

CHICAGO:

Church, Goodman and Donnelley, Steam Book and Job Printers,

108 and 110 Dearborn Street.

